# A Clinical Bacterial Dataset for Deep Learning in Microbiological Rapid On-Site Evaluation

**DOI:** 10.1038/s41597-024-03370-5

**Published:** 2024-06-08

**Authors:** Xiuli Wang, Yinghan Shi, Shasha Guo, Xuzhong Qu, Fei Xie, Zhimei Duan, Ye Hu, Han Fu, Xin Shi, Tingwei Quan, Kaifei Wang, Lixin Xie

**Affiliations:** 1 College of Pulmonary and Critical Care Medicine, Chinese PLA General Hospital, Beijing, China; 2 Chinese PLA Medical School, Beijing, China; 3 Britton Chance Center for Biomedical Photonics, Wuhan National Laboratory for Optoelectronics-Huazhong University of Science and Technology, Wuhan, Hubei 430074 China; 4 MoE Key Laboratory for Biomedical Photonics, Collaborative Innovation Center for Biomedical Engineering, School of Engineering Sciences, Huazhong University of Science and Technology, Wuhan, Hubei 430074 China

**Keywords:** Bacteria, Respiratory tract diseases

## Abstract

Microbiological Rapid On-Site Evaluation (M-ROSE) is based on smear staining and microscopic observation, providing critical references for the diagnosis and treatment of pulmonary infectious disease. Automatic identification of pathogens is the key to improving the quality and speed of M-ROSE. Recent advancements in deep learning have yielded numerous identification algorithms and datasets. However, most studies focus on artificially cultured bacteria and lack clinical data and algorithms. Therefore, we collected Gram-stained bacteria images from lower respiratory tract specimens of patients with lung infections in Chinese PLA General Hospital obtained by M-ROSE from 2018 to 2022 and desensitized images to produce 1705 images (4,912 × 3,684 pixels). A total of 4,833 cocci and 6,991 bacilli were manually labelled and differentiated into negative and positive. In addition, we applied the detection and segmentation networks for benchmark testing. Data and benchmark algorithms we provided that may benefit the study of automated bacterial identification in clinical specimens.

## Background & Summary

Lower respiratory tract infections (LRTIs) as one of the global health threats, have high morbidity and lethality in different age groups^1^. In recent years, Hospital-Acquired Pneumonia (HAP) and Ventilator-Associated Pneumonia (VAP) have a high incidence, especially in intensive care units (ICU) and have become the most common nosocomial infection in China^2^. The proliferation of hospital infection usually results in the prevalence of multidrug-resistant and extensively drug-resistant bacteria, so early and accurate etiological diagnosis is crucial for the diagnosis and treatment of infectious diseases.

Bacteria morphology identification plays an essential role in specimen interpretation for rapid bacterial identification, and it is possible to reduce the cost and yield intuitive results by visualizing microbial features through microscopy techniques. This strategy has led to the emergence and widespread use of M-ROSE^3^. During tracheoscopy, the rapid on-site smear staining technique is performed on the specimens obtained (sputum, balf or puncture tissue), and a microscope observes to obtain hints about the infectious agents, which provide critical evidence for early antibacterial agent selection^4,5^. The GRACE-VAP (Gram Stain-Guided Antibiotics Choice for VAP)^6^ program indicates that M-ROSE is not inferior to guideline-based therapy and significantly reduces the use of broad-spectrum antibiotics in ventilator-associated patients. Additionally, M-ROSE has comparable accuracy to bacterial cultures and is more than 1000 times speed than bacterial cultures^7^. A retrospective study in the ICU concluded that M-ROSE can significantly shorten the time for pathogen detection while maintaining accuracy comparable to that of clinical microbiology culture^8^. M-ROSE also plays an essential role in the initial diagnosis of cryptococcus^9^. Thus, M-ROSE has shown crucial applications in etiological diagnosis and guiding antimicrobial drug selection^10–12^.

Throughout the M-ROSE process, the identification of bacterial types and positive/negative status still relies on manual discrimination by experts with experience in microbiology. This manual identification of bacteria in M-ROSE images is time-consuming and labor-intensive. The scale of bacterial morphology is typically at the micrometre level, and revealing their morphology on slides requires imaging systems with high resolution. Generally, a complete slide imaging area is approximately 1–3 cm^2^, and after magnification by 100 times, numerous microscopic fields can be observed. Microbiologists need to transition gradually from low-power to high-power magnification, selecting areas of interest for the classification and counting of 200–500 cells and interpretation of microorganisms such as bacteria^13,14^. For higher accuracy, clinical physicians with a foundation in cytology and microbiology are recommended for these tasks. In practical clinical applications, it typically takes 2–3 hours for an experienced clinical physician to complete the counting, interpretation, and analysis of a patient’s slide sample. As a result, there is a considerable workload on clinical microbiologists, and heavy clinical work can result in relatively delayed analysis of results. Therefore, the development of an automated method for identifying bacteria in Gram-stained smear slides of lower respiratory tract specimens is a crucial aspect in enhancing the speed and quality of M-ROSE.

The advancement of deep learning affords new possibilities for bacterial recognition in M-ROSE images. However, current related research lacks the necessary support of relevant clinical data and benchmark algorithms. On one hand, numerous studies have focused on bacterial identification in culture dishes^15–18^. Some researchers found that the accuracy of automatically predicting Staphylococcus aureus (spherical or round) and Corynebacterium diphtheriae (rod-shaped) by deep learning methods exceeded 75%^19^. Hedie *et al*.^20^ developed a CNN-based platform that utilized stereo microscope images to automatically identify slime moulds, achieving an accuracy of 77.24% for genus identification and 88.92% for suborder identification. On the other hand, it is hard to find publicly available data concerning bacterial identification in clinical, hindering the development of automated identification algorithms in M-ROSE.

Here we collect Gram-stained smeared bacterial images from respiratory specimens and create their annotations for deep learning recognition in M-ROSE. In addition, we present a deep learning-based benchmark algorithm for bacterial detection and segmentation. In object detection, the YOLOv5 algorithm is applied for the classification of positive/negative cocci and positive/negative bacilli. For segmentation, the U-Net algorithm is employed to detect positive/negative classifications of bacteria. Our work can advance the application of deep learning in M-ROSE. This application contributes to the diagnosis of clinical infections and colonization and provides valuable references for treatment plan selection.

## Methods

### Ethical approval

The study was approved by the Chinese PLA General Hospital (Approval Number: 20220322001). All participants provided verbal and written informed consent. Specifically, before bronchoalveolar lavage, all patients underwent assessments to determine indications and exclude contraindications. Prior to conducting M-ROSE, patients received adequate information and provided verbal or written informed consent. Following data selection, discharged patients with M-ROSE image data were contacted by telephone to obtain verbal informed consent. In the case of patients still hospitalized, informed consent forms were signed either by the patients themselves or their legal representatives to authorize the sharing of image data obtained from bronchoalveolar lavage and M-ROSE procedures. The study conformed to the standard set by the Declaration of Helsinki, except for registration in a database.

### Data collection

We collected specimens (bronchoalveolar lavage fluid [BALF], and endotracheal aspirates) from patients undergoing bronchoalveolar lavage. Sample slides are prepared in three steps: specimen preparation, centrifugation, and smearing. After this, a Gram-stain was applied to stain the sample slides, which involved the following steps: initial staining with crystal violet, staining with iodine solution, decoloration with alcohol, and re-staining with a safranin staining solution. During ethanol decolorization, Gram-positive bacteria typically retain the purple color due to their thicker and more complex cell wall structure, which is composed of peptidoglycan, polysaccharides, phosphatic walls, and proteins, enabling them to readily absorb foreign substances. In contrast, Gram-negative bacteria have a relatively thin cell wall, and the outer lipid membrane is easily dissolved by ethanol, causing the bacteria to lose their purple color internally, and turn red during repeated staining. Occasionally, some of the dye from the initial stain remains on the periphery, giving it a dark purple color. Therefore, the Gram-stain method allows us to distinguish between Gram-positive and Gram-negative.

After preparing the sample slide, the physician will use an optical microscope (model: OLYMPUS CX31) with a 100x magnification objective lens to observe the slide and select the area of interest for imaging, instead of capturing the entire slide. A slide sample may contain several subimages, with each region having an image size of 4912 × 3684 pixels. The submitted data consisted of 1705 images from patients who underwent bedside bronchoalveolar lavage in the RICU of Chinese People’s Liberation Army (PLA) General Hospital between January 2018 and March 2022.

The dominant bacteria in these images include Gram-negative bacilli such as Acinetobacter baumannii, Klebsiella pneumoniae, Pseudomonas aeruginosa, Gram-positive bacilli such as Corynebacterium, Gram-negative cocci such as Cryptococcus, and Gram-positive cocci such as Staphylococcus aureus. Bacilli and cocci have a relatively clear morphology differentiation by shape. Gram-positive and Gram-negative organisms can be distinguished by color characteristics, with positive organisms typically bluish-violet and negative organisms typically pinkish-red, likely with slight variations due to deep staining or incomplete colorisation, and are relatively easy to identify by algorithm. The submitted data focuses only on positive/negative bacilli and cocci. Although these identifications lack subclass identification, they still contribute to guiding antibiotic treatment regimens, can reduce the use of broad-spectrum antibiotics, dynamically monitor disease changes, and assess drug efficacy.

### Data annotation

Data annotation mainly includes target detection annotation and segmentation annotation. In the target detection task, LabelImg^21^ software was used to label the images, and bacteria were divided into Gram-positive/negative coccus and Gram-positive/negative bacillus. We use a rectangular box to mark the target bacteria. We asked the rectangular box to accurately frame the boundaries of the target bacteria without excessive background components, select targets with obvious bacterial morphology, and exclude targets with missing edges or irregular morphology. For the bacterial segmentation labelling task, we used LableMe software to draw the outline of the bacteria in a manually labelled box. Judge the corresponding categories by color. Gram-positive bacteria are classified as G+ and Gram-negative bacteria are classified as G-. There are obvious differences in morphology between coccus and bacillus. Microscopically, bacteria typically have smooth edges and rounded ends, while cocci are rounded and bacilli are short rod-shaped.

**Fig. 1:**
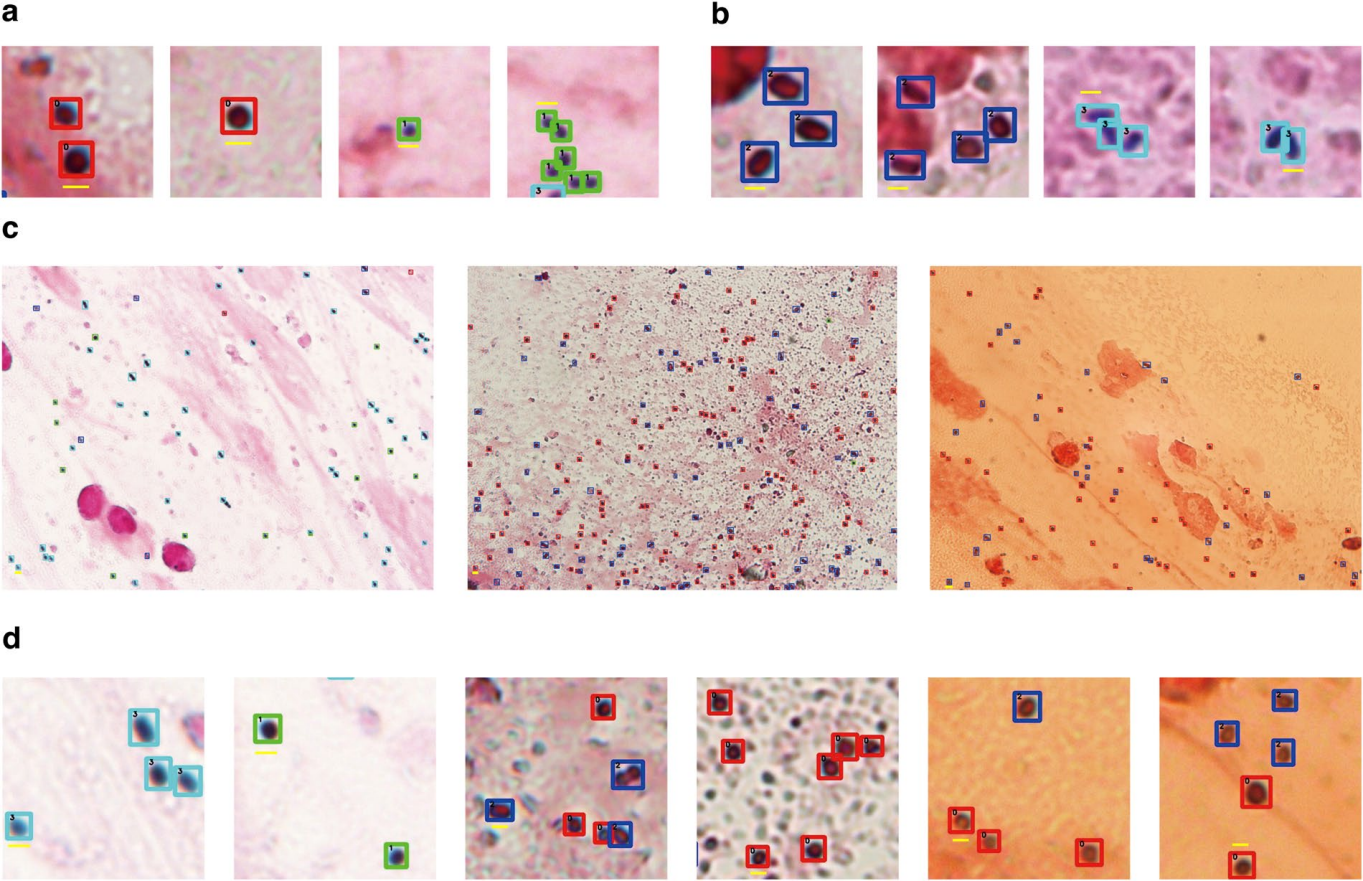
Variation analysis of different types of bacteria images in data annotation. (**a**) Example of cocci with distinctions between negative cocci (red box) and positive cocci (green box). (**b**) Example of bacilli, with divisions into positive-negative bacilli (blue box) and positive bacilli (cyan box). (**c**) The annotation results from different styles of images. (**d**) Local zoom of labelled bacteria. The scale bars (yellow line) represent 1 μm.

Fig. 1a,b respectively show typical Gram-positive/negative cocci and Gram-positive/negative bacilli. The data we collected is Gram-stained slide, for Gram-negative bacteria, Gram staining typically appears red. Due to their resilient outer membrane, ethanol faces difficulty penetrating the peptidoglycan layer to dissolve the crystal violet complex, occasionally resulting in staining as purple or developing a purple ring around the periphery (the first two panels in Fig. 1a,b). For Gram-positive bacteria, its appearance is uniformly purple in images. Because their cell walls are thick, with a higher number of layers in the peptidoglycan network, and tight cross-linking. During the decolorization process with alcohol or acetone, losing water causes the mesh to contract, additionally, the absence of lipids prevents the formation of gaps during ethanol treatment. As a result, the crystal violet and iodine complex remain firmly embedded in the cell wall, maintaining the purple color (the last two panels in Fig. 1a,b).

Differences in the shape and color of bacteria are the main basis on which we can distinguish bacteria. Fig. 1c shows images of different styles with artificial recognition; The variation in image style is mainly due to the amount of time the operator spends on staining, as well as the characteristics of the specimen. However, the features used to determine the bacteria types in Fig. 1a,b still hold in different styles of images. In other words, cocci and bacilli are distinguished primarily on the basis of morphology, while positive and negative bacteria are distinguished primarily on the basis of colour. We show in Fig. 1d a zoomed-in view of the bacterial labels of some sub-regions containing positive bacilli, positive cocci, negative cocci and negative bacilli, which can be identified by annotators with some experience. To ensure annotation accuracy, images were annotated by a total of three annotators, including a physician with extensive clinical microbiology diagnostic experience. Any discrepancies between the bacterial annotations of the two annotators were referred to the more experienced physician for determination.

## Data Records

The datasets^22^ can be categorized into three types: raw images directly collected with the micro-optical system, partially cropped images, and their annotated datasets. All of them are hosted on 10.5281/zenodo.10526360. These datasets^22^ are stored in two zip files. One zip file “RawImageDataSet” includes all raw images with a size of 18.7 Gigabytes. The other zip file is organized into 3 folders, named “640DataSet”, “DetectionDataSet” and “SegmentationDataSet”. The “640DataSet” consists of cropped images randomly extracted from their corresponding raw images. The “DetectionDataSet” and “SegmentationDataSet” consist of detection labels and semantic segmentation labels, respectively. The detection labels are standardized and stored in text files, while the segmentation label is saved in a Json file that records the shape of each bacteria as well as its positive or negative status. Training, testing and validation datasets are also included in the files “DetectionDataSet” and “SegmentationDataSet”. These datasets for training deep learning networks only include the file name. Based on the file name and its corresponding file format, we can obtain the corresponding image and its annotation.

Note that the relationship between the cropped image and the original image is indicated by the file name. For example, for a cropped image with the file name 000001_2_3 (source image name_3rd row_4th column), the first six characters denote the file name of the source image, while the subsequent characters represent different locations within that source image from which it was extracted.

## Technical Validation

### Data annotation validation

**Fig. 2:**
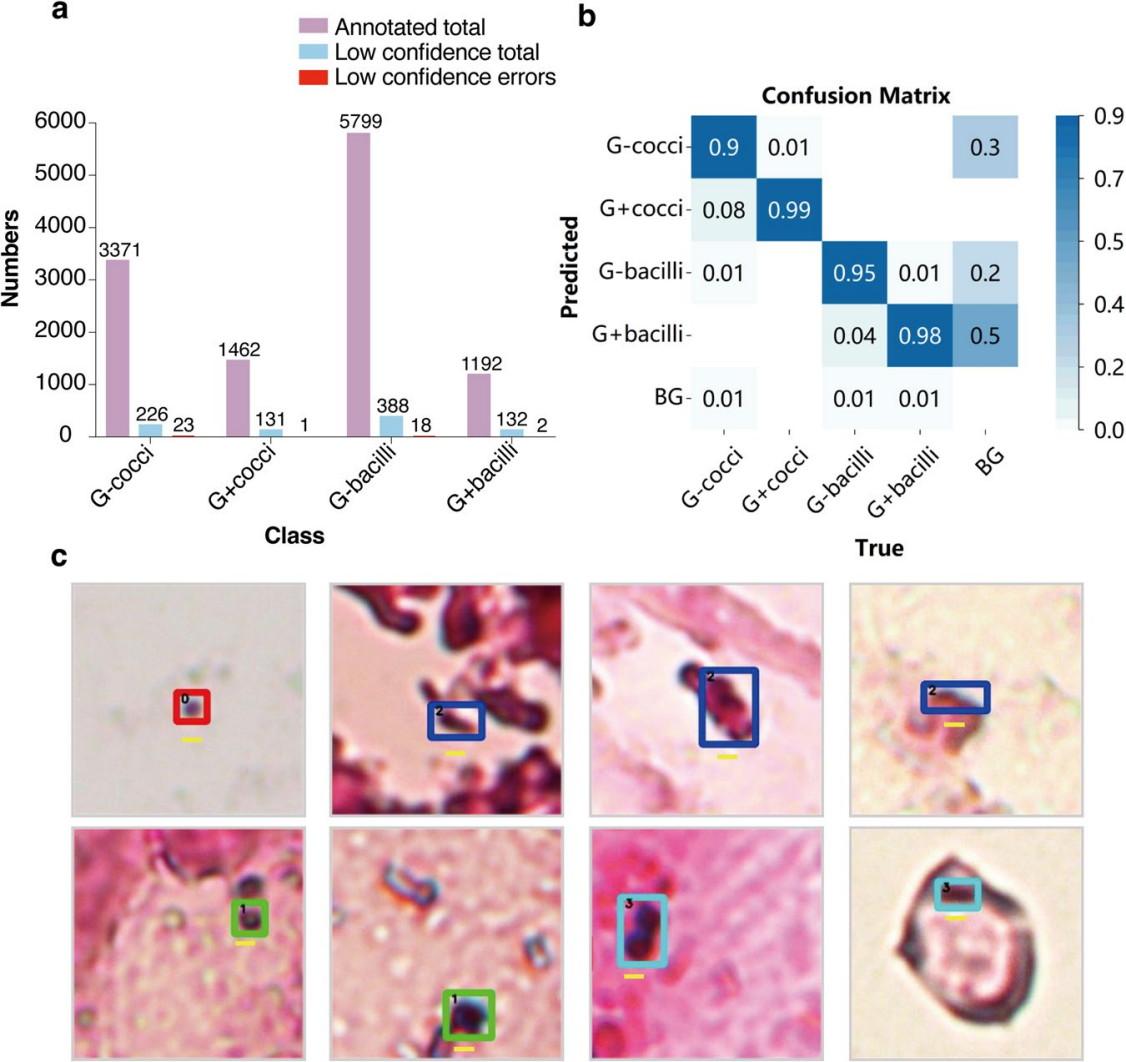
Statistics and analysis of Data annotation validation. (**a**) Total number of annotated, hard-to-annotate and re-examined inconsistent bacteria. G-cocci: Gram-negative cocci, G + cocci: Gram-positive cocci, G-bacilli: Gram-negative bacilli, G + bacilli: Gram-positive cocci. (**b**) Confusion matrix derived from rechecking the four types of hard-to-annotate bacteria. (**c**) Example of hard-to-annotation situation: Gram-negative cocci (red box); Gram-negative bacilli(blue box); Gram-positive cocci (green box). Gram-positive bacilli (cyan box).The scale bars (yellow line) represent 1 μm.

We have annotated a total of 3371 negative cocci, 1462 positive cocci, 5799 negative bacilli, and 1192 positive bacilli as shown in Fig. 2a. We trained all annotated datasets with a detection network that selects hard-to-annotate bacteria. For a bacterium, the IOU between its predicted and annotated boxes is less than 0.88. The bacterium is thought to be hard-annotated. The number of hard annotated bacteria is indicated by the pale blue bar in Fig. 2a. One of the annotators rechecked these screened bacteria and found 44 bacteria that were inconsistent with the annotated set, which are shown by red bars in Fig. 2a.

We also presented the confusion matrix for manual identification in the hard-to-annotate bacteria. In the confusion matrix, the x-axis horizontal coordinates of the matrix represent the true categories and the y-axis vertical coordinates represent the predicted categories. The values in the boxes represent the rate at which the true data is predicted to be in that category. The distribution of identification errors is summarized as follows: (1) a few errors in identifying negative and positive bacteria; (2) some bacteria with smaller morphological scales are confused with impurities in the background (Fig. 2b). Furthermore, we give some of the hard-to-annotate bacteria in Fig. 2c,where the top row shows examples of hard-to-annotate cocci and bacilli. These examples stem from the small scale of the bacteria, interference from other signals, or irregular morphology. The lower row shows examples of difficult annotations for negative and positive bacteria, mainly due to deviations of these objections from the typical features of Gram-positive or Gram-negative bacteria. From the above results, there are only a small number of inconsistent discriminants in our annotated set, which is therefore acceptable given the complexity of clinical data and the diversity of sample data.

### Deep learning networks for bacteria identification

We used two deep learning networks to validate the effectiveness of data annotation and provide the benchmark algorithms for bacterial identification in our data. The annotated datasets are split into training, validation, and test datasets according to a ratio of 7:2:1 and used for training and testing the deep learning network. In the bacterial classification of our data, the manual labels are mainly based on differences in bacterial morphology and color, and accordingly, deep learning networks should capture the differences to enable automatic identification of bacterial types with high accuracy and excellent agreement with the manual annotations. Based on this, we employ the widely used detection network and semantic segmentation network to test our labelled data. The detection network is YOLOv5 (https://github.com/ultralytics/yolov5), which detects positive/negative cocci and positive/negative bacilli in images. The segmentation network is the UNet network (https://github.com/wolny/pytorch-3dunet), which is a semantic segmentation network that separates positive and negative bacteria in an image. The loss of the detection network YOLOv5 is a weighted sum of the rectangular box loss, classification loss and confidence loss with weighting factors of 0.05, 0.5, and 1.0. The loss function for semantic segmentation is the sum of the cross entropy and Dice loss.

### Evaluation metrics

We evaluate the performance of object detection algorithms using Precision, Recall, F1score and mAP. In the calculation of these metrics, it is necessary to determine whether the target is correctly detected or not. The algorithm detection box and ground truth box with IoU > 0.45 is judged to be correctly identified. After obtaining the correctly detected targets, we compute true positive, false positive and false negative to obtain performance metrics for evaluating detection algorithms, defined as below^23,24^.

1 $${\rm{Precision}}=\frac{TP}{TP+FP}$$

2 $${\rm{Recall}}=\frac{TP}{TP+FN}$$

3 $${\rm{F1score}}=2\cdot \frac{{\rm{Precision}}\cdot {\rm{Recall}}}{{\rm{Precision}}+{\rm{Recall}}}$$

4 $$AP={\int }_{0}^{1}p(r)dr$$

5 $$mAP=\frac{{\sum }_{i=1}^{K}A{P}_{i}}{K}$$

Here, TP denotes the number of correctly detected objects, FP is the number of additional objects that do not match the ground truth, and FN is the number of objects in the ground truth that are not detected. K is the number of detected categories and p(r) refers to precision-recall curve. We still adopt the metrics in formulas (1–5) for semantic segmentation evaluation. In semantic segmentation, different values are assigned to the regions of Gram-positive and Gram-negative bacteria. If algorithmically segmented regions are dropped in annotated bounding boxes, these regions are directly binned, with each binned region representing a bacterial region. Otherwise, the region in the background region is treated as a false positive. We partition these regions into a series of connected regions, each representing a false positive bacterium. With the above operations, we can compute Precision, Recall, and F1score for bacterial semantic segmentation.

### Detection results

We manually classify bacteria into negative/positive cocci and negative/positive bacilli based on their morphological features, and accordingly, the deep learning network is also able to capture these features to achieve a fourfold classification of bacteria. Based on this, we train a detection network model to verify the effectiveness of the labelled data. We split the labelled data into a training set, a validation set, and a test set with a 7:2:1 ratio.

**Fig. 3:**
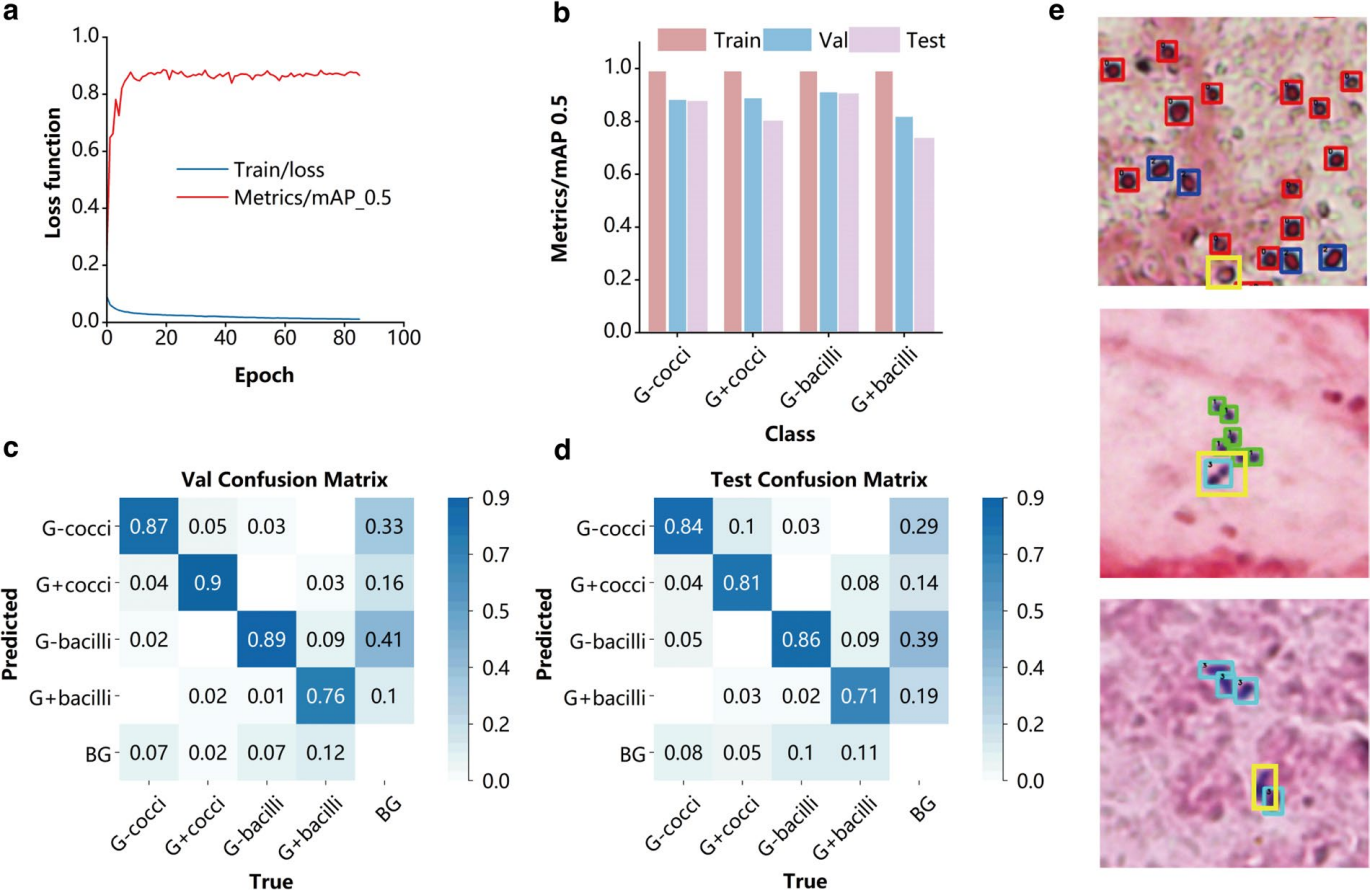
The classification performance of the detection network in analysis of annotation data. (**a**) The loss function and mean Average Precision (mAP) of YOLOv5 model during the training. (**b**) The AP of four types of bacteria classifications in YOLOv5 model. (**c**) Confusion matrix for the validation sets. (**d**) Confusion matrix for test sets. (**e**) Detection examples include some typical identification errors (yellow boxes). Top, the undetected bacterium with a certain degree of deformation. Middle, two cocci that connect and arrange in a straight line are easily misidentified as bacilli. Bottom, the bacterium whose morphology is relatively fuzzy, limited by the imaging resolution.

When the network is trained to converge, the index of the validation set reaches 0.87. This metric is a comprehensive measure of multi-category detection. This is considered correct when the overlap region between the detection box and the labelled box reaches 0.45 and they identify the same class. According to the criterion, we compute recall, precision, and mAP 0.5 scores for the training, validation, and test sets. For both validation and test sets, the mAP 0.5 score exceeds 0.73 as shown in Fig. 3b. In addition, we also present confusion matrix for the validation and test sets as shown in Fig. 3c,d.

The results show that the sources of error detections mainly include: (1) some bacteria were misidentified as background, partly due to shrinkage during the staining process; (2) some errors in identifying negative and positive bacteria. The sources of error have in common with the inconsistencies that occur in our annotations. We present some detection examples of deep learning networks. The algorithm was able to identify most of the bacteria and give the correct class, with recognition errors mainly due to mutual interference between the bacteria and some blurred edges of the bacteria. Bacterial morphology can also be affected by phagocytosis and administration of antibiotics. The presented trained deep learning networks do not give targeted solutions for these situations.

Based on the above results, we can conclude that deep learning networks can capture and detect bacterial class features in annotated data with high accuracy. Simultaneously, there is a correlation between the sources of algorithmic errors and the scenarios where annotation inconsistencies occur, confirming the effectiveness of our annotations. Moreover, it can inspire new approaches to enhance the accuracy of algorithmic detection.

### Semantic segmentation results

**Fig. 4:**
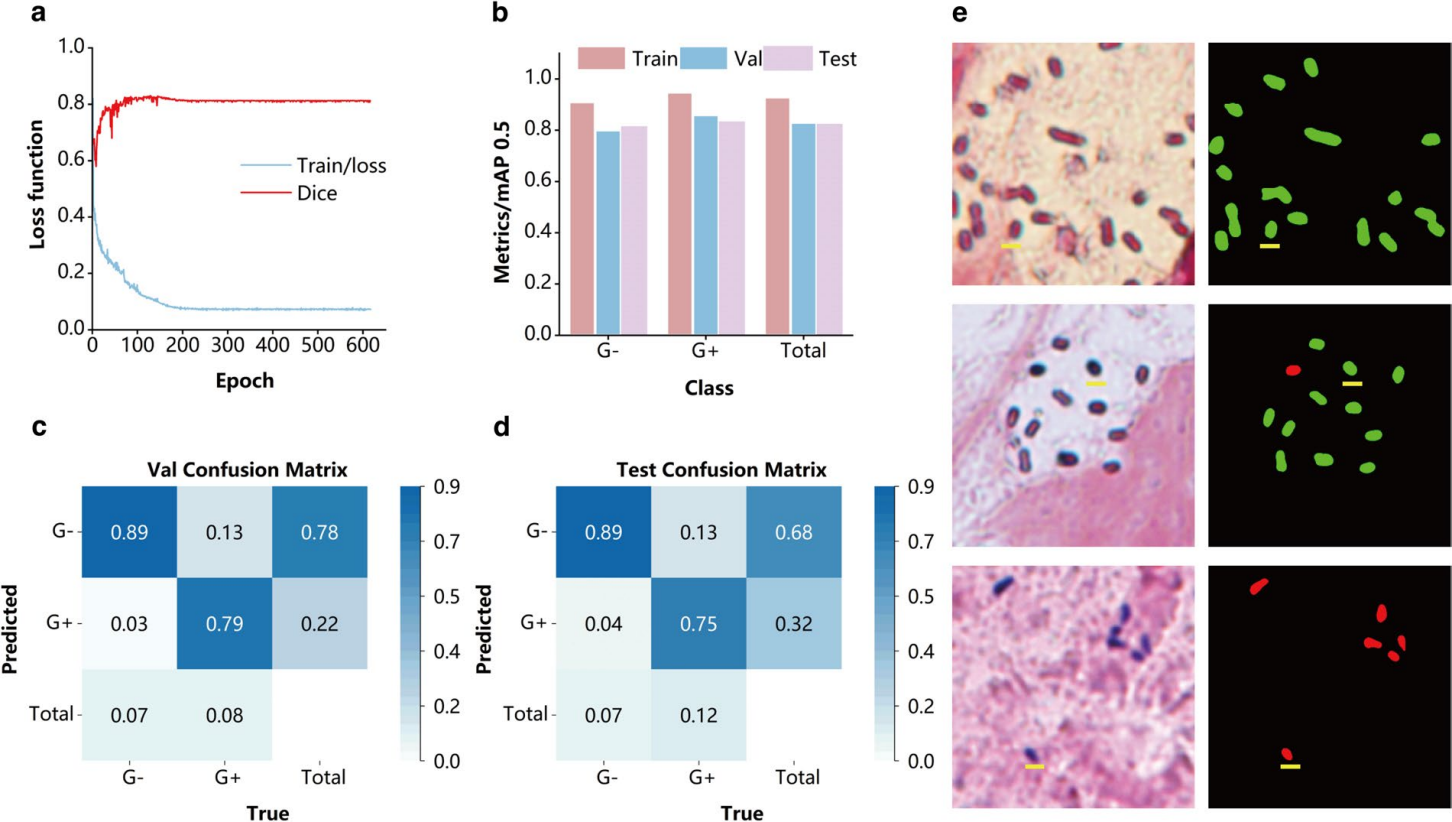
Semantic segmentation performance from annotation data. (**a**) The loss function and mean Average Precision (mAP) of U-Net model during the training. (**b**) The AP of 4 bacteria classifications in U-Net model. (**c**) Confusion matrix for the validation sets. (**d**) Confusion matrix for test sets. (**e**) Typical segmentation results of negative and positive bacteria, red for Gram-positive bacteria, green for Gram-negative bacteria.

Through our prior manual and testing validation of bacterial labelling effectiveness, we have identified some errors in the identification of both negative and positive bacteria. As a result, we have employed a semantic segmentation network to further investigate these error characteristics. The labelled set has been divided into a training set, a validation set, and a test set in a 7:2:1 ratio. Upon convergence of the network, the metrics on the validation set are 0.81, demonstrating that the semantic segmentation network is more proficient in recognizing negative and positive bacteria. The mAP values for negative and positive bacteria in the training set, validation set, and test set are presented in Fig. 4b. While these values surpass 0.8, they are still lower than the mAP values in the training set. This suggests that there are challenging cases where the algorithm struggles to recognize both negative and positive bacteria.

Through an analysis of the confusion matrix, we observed that a small percentage of bacteria are not segmented and are incorrectly identified as background, particularly some positive bacteria. These positive bacteria are more likely to be mistaken for background than negative cells due to the reduction in scale during the staining process. This reduction in scale further complicates the identification of positive cells, as their morphological characteristics become entangled with impurities in the background image, making recognition more challenging. Additionally, there is a relatively high proportion of positive bacteria incorrectly identified as negative, mainly because some positive bacteria retain red regions with complex size and distribution. These red regions require careful manual identification, posing difficulties for automatic algorithms. Fig. 4e provides typical semantic segmentation results for negative and positive bacteria. The analysis of these results reveals that the semantic segmentation network can effectively capture the features of both negative and positive bacteria, successfully identifying the majority of instances. However, there is room for improvement in the recognition accuracy of positive bacteria, necessitating enhancements in both the detection network and the semantic segmentation network.

## Usage Notes

To the best of our knowledge, this is the first publicly available clinical dataset for bacterial classification. This dataset^22^ can be used to train and evaluate different AI algorithms. In addition, the dataset and the proposed benchmark algorithms encourage developers to develop algorithms and contribute to more involvement in analyzing clinical bacteria datasets. All these datasets uploaded in Zenodo allows for use without any restriction.

### Limitations

Although the presented dataset includes a wide variety of images that took two years to collect, there are some limitations. First, all images were collected at the same hospital in Beijing, using a relatively fixed procedure. However, due to the standardized data acquisition process and diverse sample sources, the submitted data remains representative. Second, there are still some extremely dense bacteria that have not been labelled because it is difficult for experienced taggers to accurately judge the types of these bacteria in the image, especially their negative and positive cases.

## Data Availability

All codes are publicly available in the following GitHub repository https://github.com/Quanlab-Bioimage/301RoseDataSet (software licence: Apache version 2.0). The repository contains already training detection network and semantic segmentation network both of which can be used directly. Running these two networks can obtain the results presented in Figs. 3–4. The repository also contains the python codes for retraining the networks and resetting training parameters. Most of these codes are from YOLOv5 and UNet.
